# Biocompatibility and antimicrobial properties of titanium and Zirconium-infused denture base resins

**DOI:** 10.6026/9732063002001345

**Published:** 2024-10-31

**Authors:** Rajul Vivek, Sarita Aneja, Sneha Amit Rathi, Abha Kumari, Harsh Chansoriya, Shivakshi Chansoriya

**Affiliations:** 1 Department of Prosthodontics & Crown and Bridge, Purvanchal Institute of Dental Sciences, Gorakhpur, Uttar Pradesh, India; 2 Department of Dentistry, Shri Guru Ram Rai Institute of Medical Sciences and Hospital, Shri Mahant Indiresh Hospital, Dehradun, Uttarakhand, India; 3 Department of Prosthodontics & Crown and Bridge, Dr HSRSM Dental College, Hingoli, Maharashtra, India; 4 Department of Pharmacology, Rajendra Institute of Medical Sciences, Ranchi, Jharkhand, India; 5 Department of Prosthodontics & Crown and Bridge, Government College of Dentistry, Indore, Madhya Pradesh, India; 6 Department of Oral Medicine and Radiology, Government College of Dentistry, Indore, Madhya Pradesh, India

**Keywords:** Titanium, zirconium, denture base resins, biocompatibility, antimicrobial properties, polymethyl methacrylate, *Streptococcus mutans*, *Candida albicans*

## Abstract

Titanium (Ti) and zirconium (Zr) have gained attention for their promising properties in medical and dental applications, including their biocompatibility and antimicrobial effects. Denture base resins primarily made of polymethyl methacrylate (PMMA), often face issues such as microbial adhesion and limited bioactivity. Incorporating Ti and Zr into these resins could enhance their biological properties. This study investigates the biocompatibility and antimicrobial efficacy of Ti- and Zr-infused denture base resins compared to conventional PMMA resins. Three groups of denture base resins were prepared: Group A (PMMA control), Group B (PMMA + 5 wt% Ti) and Group C (PMMA + 5 wt% Zr). Biocompatibility was assessed by culturing human gingival fibroblasts (HGFs) on resin samples and performing an MTT assay to evaluate cell viability over 72 hours. Antimicrobial properties were tested against *Streptococcus mutans* and Candida albicans using a colony-forming unit (CFU) assay. Statistical analysis was performed using ANOVA with a significance level of p < 0.05. Group B (Ti-infused) exhibited a 25% increase in cell viability compared to the control group, with cell viability reaching 85% after 72 hours. Group C (Zr-infused) showed a 15% increase in cell viability. Both Ti and Zr groups demonstrated significant antimicrobial activity. Group B showed a 60% reduction in *S. mutans* CFU and a 45% reduction in *C. albicans* CFU, while Group C showed a 40% reduction in *S. mutans* and a 30% reduction in *C. albicans*. Ti- and Zr- infused denture base resins demonstrate improved biocompatibility and antimicrobial properties compared to conventional PMMA. Titanium, in particular, provides superior biological outcomes, suggesting its potential for enhancing denture base materials. These findings support the incorporation of Ti and Zr in future dental materials to improve patient outcomes.

## Background:

Denture base resins are widely used in prosthodontics, primarily composed of polymethyl methacrylate (PMMA) due to its favorable properties such as low cost, ease of manipulation and adequate aesthetics [1]. However, PMMA-based resins exhibit limitations, including poor microbial resistance and insufficient bioactivity, which can lead to oral infections such as denture stomatitis caused by *Candida albicans* and *Streptococcus mutans* [2, 3]. These infections may result in discomfort, inflammation and delayed healing in patients using dentures [4]. The incorporation of bioactive and antimicrobial agents into dental materials is an area of growing interest, aiming to improve the biological performance of these resins [5]. Titanium (Ti) and zirconium (Zr) are promising candidates due to their well-documented biocompatibility, corrosion resistance and antimicrobial properties [6, 7]. Ti and Zr have been successfully used in dental implants and prosthetic components, where they exhibit favorable interactions with biological tissues and antimicrobial effects against various oral pathogens [8]. Previous studies have indicated that Ti and Zr may improve the mechanical properties of PMMA-based resins, but their effects on biocompatibility and antimicrobial activity have not been fully elucidated [9]. Therefore, this study aims to evaluate the biocompatibility and antimicrobial properties of Ti- and Zr-infused PMMA denture base resins, hypothesizing that these modifications will enhance biological performance compared to conventional PMMA.

## Materials and Methods:

## Sample preparation:

Three groups of denture base resin samples were prepared using polymethyl methacrylate (PMMA) as the base material. Group A served as the control, consisting of conventional PMMA resin without additives. Group B consisted of PMMA resin infused with 5 wt% titanium (Ti) nanoparticles (particle size: 20-50 nm) and Group C contained PMMA resin infused with 5 wt% zirconium (Zr) nanoparticles (particle size: 30-60 nm). The Ti and Zr nanoparticles were mixed with the PMMA powder using a mechanical stirrer to ensure uniform distribution before polymerization. Each group had 10 samples, with dimensions of 10 mm x 10 mm x 2 mm, prepared following the manufacturer's guidelines for PMMA processing.

## Cell viability assay:

Human gingival fibroblast (HGF) cells were used to assess the biocompatibility of the modified resins. Cells were cultured in Dulbecco's Modified Eagle Medium (DMEM) supplemented with 10% fetal bovine serum (FBS) and 1% penicillin/streptomycin. Resin samples from each group were sterilized with 70% ethanol and placed in 24-well culture plates. HGFs were seeded onto the surface of each sample at a density of 1 x 10^4^ cells/well and incubated at 37°C with 5% CO_2_. Cell viability was assessed after 24, 48 and 72 hours using the MTT assay. Absorbance was measured at 570 nm using a microplate reader to quantify the amount of formazan produced, which correlates with cell viability.

## Antimicrobial activity:

The antimicrobial efficacy of the resin samples was evaluated against two common oral pathogens: *Streptococcus mutans* and *Candida albicans*. The bacterial (*S. mutans*) and fungal (*C. albicans*) strains were cultured in brain-heart infusion (BHI) and Sabouraud dextrose agar (SDA), respectively. Resin samples were placed in contact with bacterial and fungal suspensions (1 x 10^6^ CFU/mL) in 24-well plates and incubated at 37°C for 24 hours.

After incubation, each sample was removed, washed with phosphate-buffered saline (PBS), and the remaining adherent microorganisms were detached by sonication. The suspension was then serially diluted and spread onto agar plates for CFU counting. The number of viable colonies was quantified after 24 hours of incubation, and the reduction in CFU was calculated by comparing the test groups (Ti- and Zr-infused resins) to the control group (PMMA).

## Statistical analysis:

All experiments were conducted in triplicate. Data were presented as mean ± standard deviation (SD). Statistical analysis was performed using one-way ANOVA followed by Tukey's post-hoc test to determine significant differences between groups. A p-value < 0.05 was considered statistically significant.

## Results:

## Cell viability (MTT assay):

The results of the MTT assay for cell viability at 24, 48 and 72 hours are shown in Table 1. Group B (PMMA + Ti) demonstrated significantly higher cell viability compared to the control (Group A) and Group C (PMMA + Zr) at all-time points. After 72 hours, Group B showed 85% cell viability, a 25% increase compared to Group A (60%). Group C displayed a moderate increase in cell viability, reaching 70% at 72 hours.

## Antimicrobial activity (CFU assay):

The antimicrobial activity results for *Streptococcus mutans* and *Candida albicans* are summarized in Table 2. Both Group B (PMMA + Ti) and Group C (PMMA + Zr) demonstrated significant antimicrobial effects compared to Group A (Control). Group B exhibited the greatest reduction in *S. mutans* (60%) and *C. albicans* (45%) colony counts. Group C also showed antimicrobial effects, with a 40% reduction in *S. mutans* and a 30% reduction in *C. albicans*.

## Statistical analysis:

One-way ANOVA followed by Tukey's post-hoc test revealed significant differences between Group B (PMMA + Ti) and Group A (Control) in both cell viability and antimicrobial activity (p < 0.05). Group C (PMMA + Zr) also showed significant improvements compared to Group A, but Group B performed better in both biocompatibility and antimicrobial tests (p < 0.05). These results indicate that titanium infusion in PMMA provides superior biological outcomes, with enhanced cell viability and stronger antimicrobial effects, compared to zirconium infusion and the control group.

## Discussion:

The findings of this study demonstrate that the incorporation of titanium (Ti) and zirconium (Zr) nanoparticles into PMMA-based denture resins significantly improves both biocompatibility and antimicrobial properties. These enhancements address two common concerns in denture base resins: poor biological interaction with oral tissues and susceptibility to microbial colonization.

The increase in cell viability observed in the Ti- and Zr-infused resin groups, particularly in the Ti group (85% after 72 hours) and suggests that these materials are more conducive to fibroblast adhesion and proliferation compared to conventional PMMA [1]. Titanium's well-established biocompatibility is likely a result of its favorable surface characteristics, such as its ability to form a stable oxide layer, which promotes cell attachment and differentiation [2, 3]. Several studies have shown similar outcomes where titanium-containing dental materials support enhanced cell growth and viability [4, 5]. The moderate improvement in biocompatibility observed with Zr may be attributed to its good tissue compatibility and corrosion resistance, though it appears to be less bioactive than Ti in the context of denture base resins [6].

Both Ti- and Zr-infused resins exhibited significant antimicrobial effects, with the Ti group showing a 60% reduction in *Streptococcus mutans* and a 45% reduction in *Candida albicans*. These results align with previous research demonstrating the antimicrobial capabilities of titanium nanoparticles, which are thought to disrupt bacterial cell membranes and inhibit biofilm formation [7, 8]. The antimicrobial effect of Zr was also notable, albeit slightly lower than Ti, with a 40% reduction in *S. mutans* and 30% in *C. albicans*. Zirconium oxide has been studied for its antimicrobial properties, though its mechanisms are less understood compared to titanium [9].

The antimicrobial properties of Ti and Zr in this study are particularly relevant for preventing denture-related infections such as denture stomatitis, which is often associated with microbial biofilm formation on PMMA surfaces [10, 11]. The ability to reduce microbial adhesion and growth without the need for additional chemical disinfectants could significantly improve the clinical performance of denture base materials [12].

Previous studies on Ti- and Zr-doped dental materials support the findings of this study. For instance, a study by Alrahlah *et al.* [5] demonstrated enhanced mechanical properties and antifungal effects when TiO^2^ nanoparticles were incorporated into PMMA. Similarly, Patel *et al.* [13] found that ZrO^2^ nanoparticles improved the flexural strength of PMMA, though the antimicrobial properties were not as pronounced as those of TiO^2^. The current study further confirms the superior antimicrobial performance of Ti-infused PMMA over Zr-infused variants [14].

Interestingly, the biocompatibility and antimicrobial performance of Ti-doped materials in this study are comparable to some of the newer bioactive materials in the field of dentistry, such as calcium phosphate-based cements and glass ionomers, which have been designed to promote tissue healing and prevent microbial infections [15]. However, the ease of integration and cost-effectiveness of Ti and Zr nanoparticles make them attractive candidates for widespread use in denture base resins. The improvement in both biocompatibility and antimicrobial activity suggests that Ti-infused PMMA resins could provide enhanced clinical outcomes for denture wearers, particularly those prone to microbial infections or poor tissue compatibility [7]. Moreover, the use of Zr, while not as potent as Ti in this study, may still be beneficial for patients with specific metal sensitivities, as zirconium is known for its hypoallergenic properties [6]. Among all tested bacterial strains, biofilms grown on the zirconium nitride surface exhibited a higher proportion of dead bacteria compared to other disks. While the titanium nitride surface also demonstrated the ability to inactivate bacterial biofilms, its effectiveness was comparatively lower [16]. Further studies could explore the long-term performance of these materials *in vivo*, as well as their mechanical properties under dynamic oral conditions. This study had several limitations, including its *in vitro* nature, which may not fully replicate the complex oral environment where various factors such as saliva, temperature changes and mechanical forces can influence material performance. Additionally, the antimicrobial effects were tested against two specific microorganisms, but other oral pathogens may also play a role in denture-related infections [10]. Future studies should include a wider range of microbial species and test these materials under more clinically relevant conditions.

## Conclusion:

The results of this study demonstrate that Ti- and Zr-infused denture base resins offer significant improvements in biocompatibility and antimicrobial properties compared to conventional PMMA. Titanium, in particular, provides superior biological performance, making it a promising candidate for enhancing the clinical efficacy of denture base materials. These findings suggest that incorporating bioactive nanoparticles such as Ti and Zr into dental materials could improve patient outcomes by reducing the risk of infections and promoting better tissue integration.

## Figures and Tables

**Table 1:** Cell Viability (% of Control) at 24, 48 and 72 Hours

**Time (Hours)**	**Group A (Control)**	**Group B (PMMA + Ti)**	**Group C (PMMA + Zr)**
24	55 ± 3%	70 ± 4%	60 ± 3%
48	58 ± 2%	80 ± 3%	65 ± 4%
72	60 ± 4%	85 ± 5%	70 ± 4%

**Table 2:** Reduction in Colony Forming Units (CFU) Compared to Control

**Microorganism**	**Group A (Control)**	**Group B (PMMA + Ti)**	**Group C (PMMA + Zr)**
*S. mutans*	Baseline (100%)	60 ± 3% reduction	40 ± 5% reduction
*C. albicans*	Baseline (100%)	45 ± 4% reduction	30 ± 4% reduction
